# Investigating potential links between gut microbiome, clinical parameters, and mortality in long-living male patients receiving multi-drug therapy

**DOI:** 10.3389/fcimb.2025.1456794

**Published:** 2025-07-07

**Authors:** Quanxin Wu, Hui Pan, Xianghua Zeng, Fei Xiong, Yongling Lv, Zhiwei Jiang, Jiahuan Li, Li Wang, Xiao Wang

**Affiliations:** ^1^ Second Division of Cadre Ward, General Hospital of Central Theater Command, Wuhan, China; ^2^ Department of Medical Oncology, Chongqing University Cancer Hospital and Chongqing Cancer Institute, Chongqing, China; ^3^ Department of Radiology. General Hospital of Central Theater Command, Wuhan, China; ^4^ Maintainbiotech Ltd., Wuhan, China

**Keywords:** intestinal microbiome, longevity, multimorbidity, polypharmacy, mortality

## Abstract

**Background and aims:**

Multimorbidity and polypharmacy are common in long-living patients and have been linked to alterations in gut microbiota. However, the prognostic relevance of these microbiota changes remains unclear in this population. We hypothesized that clinical parameters and gut microbial profiles are associated with survival outcomes. This exploratory study aimed to investigate the relationships between intestinal microbiota, clinical variables, and 18-month mortality in long-living male patients undergoing multi-drug therapy.

**Methods:**

33 long-living male adults (mean age 94 ± 4 years) were enrolled. Upon admission, clinical parameters and stool samples were collected. The composition of intestinal microbiome was examined using 16S rRNA gene sequencing technology. Subsequently, the patients were categorized into death and no-death groups based on their survival status after 18 months. Risk factors associated with 18-month mortality in patients were evaluated.

**Results:**

The 18-month mortality rate was 48.5%. In this exploratory analysis, higher CIRS-G scores and creatinine levels were independently associated with mortality (HR: 1.323 and 1.007, respectively). CIRS-G had high prognostic value (AUC = 0.967). IL-6 levels and renal/hepatic/pancreatic comorbidities were also elevated in the death group. Notably, *Erysipelatoclostridium* was enriched in survivors and *Sutterella* in non-survivors, with correlations to clinical parameters.

**Conclusions:**

CIRS-G scores and serum creatinine levels demonstrated potential predictability for prognostic value in forecasting mortality among long-living male patients. Moreover, a potential association between CIRS-G score and intestinal microbiome was observed. These results underscore the intricate impact of comorbidities and polypharmacy on intestinal microbiome composition and clinical consequences. Clinically, the CIRS-G score could serve as a valuable tool for identifying high-risk elderly patients who may benefit from closer monitoring and individualized interventions. However, the small sample size, monocentric design, and potential confounders limit generalizability. These findings support further validation in larger cohorts.

## Introduction

With the advancements in medical technology and improved sanitation, there has been a significant increase in human life expectancy. It is projected that by 2050, the global population aged 65 years or older will reach 1.6 billion (Harper, 2014; Santoro et al., 2018; Singh et al., 2019). Consequently, the issue of multi-disease coexistence and multi-drug treatment in senior adults has gained prominence, emerging as a critical challenge in 21st-century healthcare. Multi-disease coexistence refers to the presence of more than two chronic diseases in an individual, such as hypertension, diabetes, coronary heart disease, cancer, or metabolic disorders commonly observed in senior adults (Vaiserman et al., 2017; Kong et al., 2019). The prevalence of multi-disease coexistence is becoming more common in high-income countries and is rapidly increasing in low- and middle-income countries, posing a significant public health concern (Salive, 2013; Arokiasamy et al., 2015; MacMahon et al., 2018; Hu et al., 2022). Individuals with multi-disease coexistence often require multi-drug therapy, which can lead to challenges such as drug interactions, adverse drug reactions, and antibiotic resistance, ultimately complicating treatment and exacerbating health issues (Nagata et al., 2022; Gemikonakli et al., 2023). In developed countries, multi-drug therapy is prevalent due to the availability of drugs and adherence to treatment guidelines, contributing to the rise in multi-drug use (Guillot et al., 2020; Pazan and Wehling, 2021; Cole et al., 2023). The presence of multi-disease coexistence and the necessity for multi-drug therapy not only impact the quality of affected individuals but also impose a substantial burden on families and society.

The intestinal microbiome constitutes a vast and intricate community of microorganisms within the human gastrointestinal tract, encompassing bacteria, fungi, viruses, and protozoa (Valdes et al., 2018). The composition and functionality of the intestinal microbiome are influenced by genetic factors, such as individual genotype, and epigenetic factors, including maternal microflora, diet, lifestyle, disease, and antibiotic therapy (Bäckhed et al., 2015; Loh et al., 2015; Massari et al., 2019). A healthy gut micro-ecosystem plays a crucial role in preserving the metabolic, immune, and neuroendocrine equilibrium of the host (McSweeney et al., 2019; Franzin et al., 2020; Zhou et al., 2020). Nevertheless, the diversity and resilience of the intestinal microbiome decline with age, particularly in the senior population (Ahmed et al., 2010; Yatsunenko et al., 2012; Biagi et al., 2016; Ling et al., 2020). The alterations in intestinal microbiome are influenced by numerous factors and exhibit variations across different research studies. Generally, in comparison to young adults, the senior population shows a significant increase in the levels of *Enterobacterium* and *Enterococcus* in the gut, while the levels of *Bifidobacterium* decrease. At the phylum level, there is an elevated relative abundance of Firmicutes and Actinobacteria in the senior population, accompanied by a reduced relative abundance of Bacteroidetes. These changes result in an increased Firmicutes/Bacteroidetes ratio. However, with advancing age, this ratio may decrease in certain senior individuals. Furthermore, there is no consensus regarding age-related changes in Proteobacteria (Ahmed et al., 2010; Biagi et al., 2016; Kong et al., 2016; Salazar et al., 2017; Kong et al., 2019). Alterations in the intestinal microbiome could be intricately linked to nutrient absorption, immune modulation, and inflammatory reactions. This relationship is particularly significant in the senior demographic, where a decline in the richness and resilience of the intestinal microbiome might disturb its equilibrium, consequently contributing to the onset and progression of diverse diseases.

The impact of diseases and medications on intestinal microbiome has been widely documented (Rondanelli, 2015). Certain diseases can directly influence the intestinal microbiome. For instance, individuals with diabetes and inflammatory bowel disease (IBD) often exhibit reduced intestinal microbiome diversity, characterized by a decline in beneficial bacteria like *Bifidobacterium* and *lactobacillus*, and an increase in pathogenic bacteria such as *Enterobacterium* and other potential pathogens (Karlsson et al., 2013; Mentella et al., 2020; Lau et al., 2021). Drug therapy is also known to significantly modify the composition of intestinal microbiome. Antibiotic usage, for example, can lead to a reduction in beneficial bacteria and an increase in resistant strains, while prolonged use of proton pump inhibitors (PPIs) may elevate the risk of intestinal infections (Vich Vila et al., 2020; Weersma et al., 2020). Statins, a class of drugs primarily prescribed for lowering cholesterol levels, can indirectly impact intestinal microbiome composition by influencing bile acid metabolism (She et al., 2024). Importantly, these alterations are not permanent, as the balance of intestinal microbiome can be partially restored through dietary adjustments, probiotic supplementation, prebiotics, or other interventions, thereby enhancing patient health. For instance, prebiotics like inulin and *fructooligosaccharides* have been shown to stimulate the growth of *Bifidobacterium* and *Lactobacillus*, thus promoting a healthier intestinal microbiome balance (Claus, 2017; Tandon et al., 2019). Moreover, specific dietary approaches, such as the Mediterranean diet have been found to enhance intestinal microbiome diversity and increase the abundance of beneficial bacteria (Merra et al., 2020). The damage caused by disease and multi-drug treatment is not inevitable with age. Research indicates that certain microbial taxa like *Coriobacteriaceae*, *Oxalobacter*, and the probiotic *Lactobacillus amylovorus* may be linked to extended lifespan. In a study involving senior hospitalized patients, polypharmacy was significantly correlated with reduced species richness of intestinal microbiome, and alterations in intestinal microbiome were associated with mortality increased during follow-up (Ticinesi et al., 2017). The intestinal microbiome composition in long-lived individuals with multiple comorbidities and polypharmacy exhibits unique characteristics that hold significant physiological and pathological implications. Understanding the intricate interplay between disease, drug therapy, and intestinal microbiome is crucial for devising effective intervention strategies that could potentially aid in the prevention and management of geriatric syndromes (Gemikonakli et al., 2021; Victoria et al., 2022).

Numerous studies have delved into the impacts of age, disease, and medications on the intestinal microbiome. However, limited research has explicitly focused on long-living male patients-an understudied demographic with unique biological and clinical characteristics. This cohort often presents with high disease burdens and complex medication regimens, resulting in a distinctive interaction between host factors and the intestinal microbiome. Furthermore, while polypharmacy and multimorbidity are common in geriatric research, the specific interplay between these elements in long-lived males remains inadequately characterized in the literature. The objective of this study was to analyze the impact of multimorbidity and polypharmacy on mortality in elderly male patients, as well as the association between these factors and gut microbiota, and to further understand the changes in gut microbiota in this special population and its potential impact on 18-month clinical outcomes. Utilizing 16S rRNA gene sequencing, we scrutinized the characteristics of intestinal microbiota in 33 long-lived male patients with concurrent multiple diseases and polypharmacy and evaluated the relationship between pertinent factors and clinical outcomes over an 18-month period. The objective was to gain deeper insights into the intestinal microecological characteristics and significant clinical risk factors in long-lived male patients, and to offer potential strategies for enhancing clinical management and ameliorating prognosis.

## Materials and methods

### Study design and sample collection

An observational study design was employed to recruit long living male patients (aged ≥85 years) with multiple chronic diseases (≥3 chronic diseases) who were admitted to the geriatric department of General Hospital of Central Theater Command from October 2021 to June 2022. Exclusion criteria were as follows:1) Concurrent with infectious diseases (such as bacterial pneumonia, viral hepatitis, gastrointestinal inflammation, gallbladder pancreatitis or other inflammatory diseases), acute or chronic gastrointestinal disorders (intestinal ischemia, intestinal cancer, cirrhosis, constipation, inflammatory bowel disease, or a history of abdominal surgery within 6 months), undergoing hemodialysis, or respiratory failure requiring mechanical ventilation; 2) Recent use of antibiotics or probiotics within the past month; 3) History of smoking or alcohol consumption.

Clinical metadata, including age, weight, BMI, primary diagnosis, comorbidities, and type of medication, were collected in accordance with the core elements of the Minimum Information about a Microbiome Study (MIMs) guidelines. Although not all items in the MIMs framework were applicable or feasible due to the specific population and study context, we strived to ensure adequate documentation of sample-associated variables to facilitate reproducibility and data comparability. Upon admission, laboratory tests were conducted, including blood routine, CRP, IL-6, liver and kidney function, electrolyte levels, blood glucose, lipid profile, stool routine and urine analysis, and fresh stool samples were obtained. The health status of the participants was assessed using the Cumulative Illness Rating Scale for Geriatrics (CIRS-G) (Salvi et al., 2008), which evaluates 14 organ systems on a scale of 0 to 4, resulting in an overall score ranging from 0 to 56. Multi-drug therapy was assessed by identifying the long-term medications taken by each patient. Follow-up was conducted for 18 months to record the survival status of all patients.

Due to the observational nature of the study and the specific characteristics of the elderly inpatient population, each patient provided a single stool sample at the time of hospital admission. No technical replicates or biological replicates were performed. However, strict protocols were followed for sample collection, preservation, and DNA extraction to minimize technical variability. All samples were processed in a standardized manner to ensure consistency and reliability of the microbiome profiling.

### DNA library preparation and 16S rRNA sequencing

Fecal samples were obtained within 24 hours of admission and all fresh fecal samples were placed in sterile containers and frozen at -80°C for 24 hours until DNA extraction. PCR reaction conditions: predenaturation at 95°C for 3 min; denaturation at 95°C for 30 s, annealing at 55°C for 30 s, and extension at 72°C for 15 s, for 25 cycles; final extension at 72°C for 5 min, then hold at 4°C. All reagents were from the same lot (HiPure Stool DNA Kit, Cat No: D314103, Magen, Guangzhou, China). No missing data were imputed. Outliers in quantitative variables were identified by ± 1.5 IQR rule and retained unless attributable to procedural error. PCR products were detected by agarose gel electrophoresis. Amplified DNA libraries were purified using AMPure XP microbeads (Beckman Coulter, Fullerton, CA, United States), and library product concentrations were quantified using Qubit. Following the validation of the library, amplicon sequencing was performed on the Illumina Novaseq6000 platform using a paired-end 250 bp strategy. The DADA2 software package (v1.28.0) in R language (version 4.2) was used for denoising. DADA2 employs a set of error estimation models to infer the true sequence before the introduction of incorrect bases in PCR amplification and sequencing. In addition, quality control was performed by filtering sequences based on length, mean quality scores, and removing low-quality sequences. The sequencing depth was determined by rarefaction analysis, ensuring sufficient coverage for diversity analysis.

### Bioinformatics and statistical analysis

Bacterial 16S rRNA sequences were analyzed utilizing the QIIME2 (v2021.8) (Bolyen E et al., 2019) software. Denoising of the sequences was conducted through the application of the DADA2 (Herlemann et al., 2011) software package within the R programming language. The DADA2 tool employs error estimation models to deduce the accurate sequence prior to the introduction of erroneous bases during PCR amplification and sequencing. Subsequently, amplicon sequence variants (ASVs) were inferred using the DADA2 pipeline, which utilizes a higher-resolution method for sequence variant determination. The 16S rRNA sequences were sourced from the SILVA 138.1 database, The QIIME2 (Bolyen E et al., 2019) suite was employed for ASV classification and determination of the relative abundance of each taxon across all samples. The QIIME2 plug-in feature-classifier fit-classifier-naive bayes (Bokulich et al., 2018) was utilized to generate a bar graph illustrating the species distribution, followed by the application of the QIIME2 plug-in feature-classifier classify-sklearn to annotate the feature sequences. The bar chart displayed the top 10 taxa in terms of abundance. The *α*-diversity of gut microbiota (using Shannon index) was compared by Wilcoxon test, Bray-curtis distance based Principal Coordinate Analysis (PCoA) was performed for microbial *β*-diversity analysis (using Adonis analysis). PCoA analysis was conducted using QIIME2 (Caporaso et al., 2010), and the PCoA diagram was generated using the R package ggplot2 (v3.4.2). PERMANOVA (Adonis test in the vegan package v2.6-2) was used to assess differences in beta diversity across groups, providing a multivariate analysis of variance based on dissimilarity matrices. Linear discriminant analysis (LDA) effect size (LefSe) method was used to identify the different bacteria genera between the death group and the no-death group, and Wilcoxon test was employed to detect strains with a significance level of *p*<0.05. Bacteria that exhibited significant differences with LDA scores ≥2.0 were visually represented on taxonomic bar plots.

All statistical analyses were conducted using SPSS version 23.0 (IBM Corp., Armonk, NY, USA). For continuous variables with a normal distribution, comparisons between two groups were performed using the independent sample t-test. The Wilcoxon test was employed for non-parametric data that did not meet the assumptions of normality. Categorical variables were analyzed using the Chi-square test or Fisher’s exact test, as appropriate. In order to identify potential factors associated with mortality at 18 months, univariate Cox proportional hazards regression analysis was initially performed. Subsequently, a multivariate Cox regression model was constructed using stepwise selection to determine significant predictors, which was also implemented in SPSS 23.0. The prognostic performance of the identified factors was evaluated by calculating the area under the curve (AUC) of receiver operating characteristic (ROC) curves, with analyses conducted using MedCalc Statistical Software version 22.0 (MedCalc Software Ltd., Ostend, Belgium). A larger AUC value indicates better discrimination ability in predicting prognosis. The results obtained were then used to develop a nomogram to predict 12-month and 24-month mortality rates using R software. To explore the impact of environmental factors on gut microbiota differences between groups, which might influence 18-month mortality, redundancy analysis (RDA) was performed. The RDA and subsequent plotting were completed using the ggvegan package (v0.1.0) and ggplot2 package (v3.4.2) within the R programming environment. The significance levels for tests were defined as *p*<0.05 (*) and *p*<0.01 (**).

## Results

### Clinical characteristics of patients

A total of 33 hospitalized patients were enrolled in the study. None of the patients had a history of smoking or drinking, and their daily dietary patterns remained relatively stable. The average age of the patients was 94 ± 4 years. The clinical characteristics of the participants are detailed in **Table 1**. All patients exhibited a high burden of chronic illnesses, as indicated by CIRS-G scores ranging from 16 to 33. After an 18-month follow-up period, it was observed that the CIRS-G scores were significantly higher in the death group compared to the no-death group (*p*<0.01). The most prevalent reasons for hospitalization included coronary heart disease/arrhythmia, hypertension, chronic bronchitis/COPD, stroke, dementia, diabetes, and renal/liver/pancreatic diseases. While there was no notable difference in the number of chronic comorbidities between the two groups, the prevalence of renal/liver/pancreatic diseases was higher in the death group (*p*<0.05). All patients were subjected to multi-drug therapy (involving the long-term use of ≥5 medications). Commonly prescribed drugs included statins (84.4%), vitamin D supplements (69.7%), prostatic hyperplasia agents (60.6%), ACEI/ARB (57.6%), PPIs (54.5%), antiplatelet aggregation agents (48.5%), and dihydropyridine derivatives (48.5%). Although there was no significant difference in the number of medications between the no-death and death groups, the utilization rate of ACEI/ARB was higher in the no-death group (*p*<0.05). Furthermore, IL-6 levels were found to be elevated in the death group compared to the no-death group (*p*<0.05).

**Table 1:** Demographic characteristics of the volunteers who participated in the study.

Variable	Total (n=33)	No-Death (n=17)	Death (n=16)	*p* value	*adjusted p*
Age (years) (mean ± SD)	94 ± 4	94 ± 4	94 ± 4	0.821	1
BMI (kg/m^2^) (mean ± SD)	20.20 ± 1.58	20.30 ± 1.43	20.09 ± 1.77	0.710	1
CIRS-G Score (mean ± SD)	23 ± 5	20 ± 2	27 ± 4	<0.001	<0.001
White Blood Cells (mean ± SD)	6.78 ± 2.08	7.11 ± 2.13	6.44 ± 2.04	0.370	0.905
CRP (mg/dL) (median and IQR)	0.50 [0.50-5.81]	0.50 [0.50-0.81]	1.75 [0.50-7.13]	0.131	0.617
IL-6 (pg/ml) (median and IQR)	11.2 [7.1-14]	8.7 [6.6-13]	13.25 [10.1-20.45]	0.034	0.293
Hemoglobin (g/L) (mean ± SD)	111.88 ± 19.86	112.29 ± 17.72	114.44 ± 22.49	0.904	1
Serum glucose (mmol/L) (median and IQR)	5.98 [5.31-7.25]	6.63 [5.69-7.31]	5.70 [5.11-6.48]	0.759	1
Creatinine (μmol/L) (median and IQR)	77 [62–97]	74 [56-94]	89 [67-121]	0.144	0.617
Total cholesterol (mmol/L) (mean ± SD)	3.38 ± 0.61	3.31 ± 0.60	3.47 ± 0.63	0.457	0.979
Triglycerides (mmol/L) (mean ± SD)	1.10 ± 0.47	1.13 ± 0.43	1.06 ± 0.52	0.691	1
Comorbidities, n (%)					
Number of chronic comorbidities (median and IQR)	5 [4-6]	5 [4-5]	6.5 [4-7]	0.068	0.408
Dementia	21 (63.6%)	10 (58.8%)	11 (68.8%)	0.554	1
Hypertension	29 (87.9%)	15 (88.2%)	14 (87.5%)	0.948	1
Diabetes mellitus	14 (42.4)	8 (47.1%)	6 (37.5%)	0.579	1
Respiratory diseases	24 (72.7%)	12 (70.6%)	12 (75.0%)	1.00	1
Cardio cerebral vascular diseases ^*^	29 (87.9%)	15 (88.2%)	14 (87.5%)	1.00	1
Renal/liver/pancreatic diseases	7 (21.2%)	1 (5.9%)	6 (37.5%)	0.039	0.293
Drugs, n (%)					
Number of drugs (n, mean ± SD)	15 ± 4	15 ± 3	16 ± 5	0.844	1
DBI (median and IQR)	0 [0-0]	0 [0-0]	0 [0-1]	0.363	0.905
Statins	28 (84.8%)	15 (88.2%)	13 (81.3%)	0.656	1
Proton pump inhibitors	18 (54.5%)	9 (52.9%)	9 (56.3%)	0.849	1
Platelet aggregation inhibitors	16 (48.5%)	8 (47.1%)	8 (50.0%)	0.866	1
ACEI/ARB	19 (57.6%)	13 (76.5%)	6 (37.5%)	0.024	0.293
Beta blockers	12 (36.4%)	5 (29.4%)	7(43.8%)	0.392	0.945
Dihydropyridine derivatives	16 (48.5%)	10 (58.8%)	6 (37.5%)	0.221	0.678
Hyperplasia of prostate inhibitor	20 (60.6%)	12 (70.6%)	8 (50.0%)	0.226	0.678
Vitamin D	23 (69.7%)	11 (64.7%)	12 (75.0%)	0.708	1
Metformin	4 (12.0%)	2 (11.8%)	2 (12.5%)	1.0	1

*p* values are for comparison between Death and No-Death patients.

*p* value was adjusted with the FDR Benjamini-Hochberg in account for multiple tests.

Threshold for statistical significance: *p* = 0.05.

*Cardio and neurological diseases other than dementia.

BMI, body mass index; CIRS-G, cumulative illness rating scale-geriatric; CRP, c-reactive protein; IL-6, interleukin 6; DBI, drug burden index; ACEI, angiotensin converting enzyme inhibitor; ARB, angiotensin II receptor blocker.

### Examination of the distribution and diversity of intestinal microbiome species

**Figure 1:**
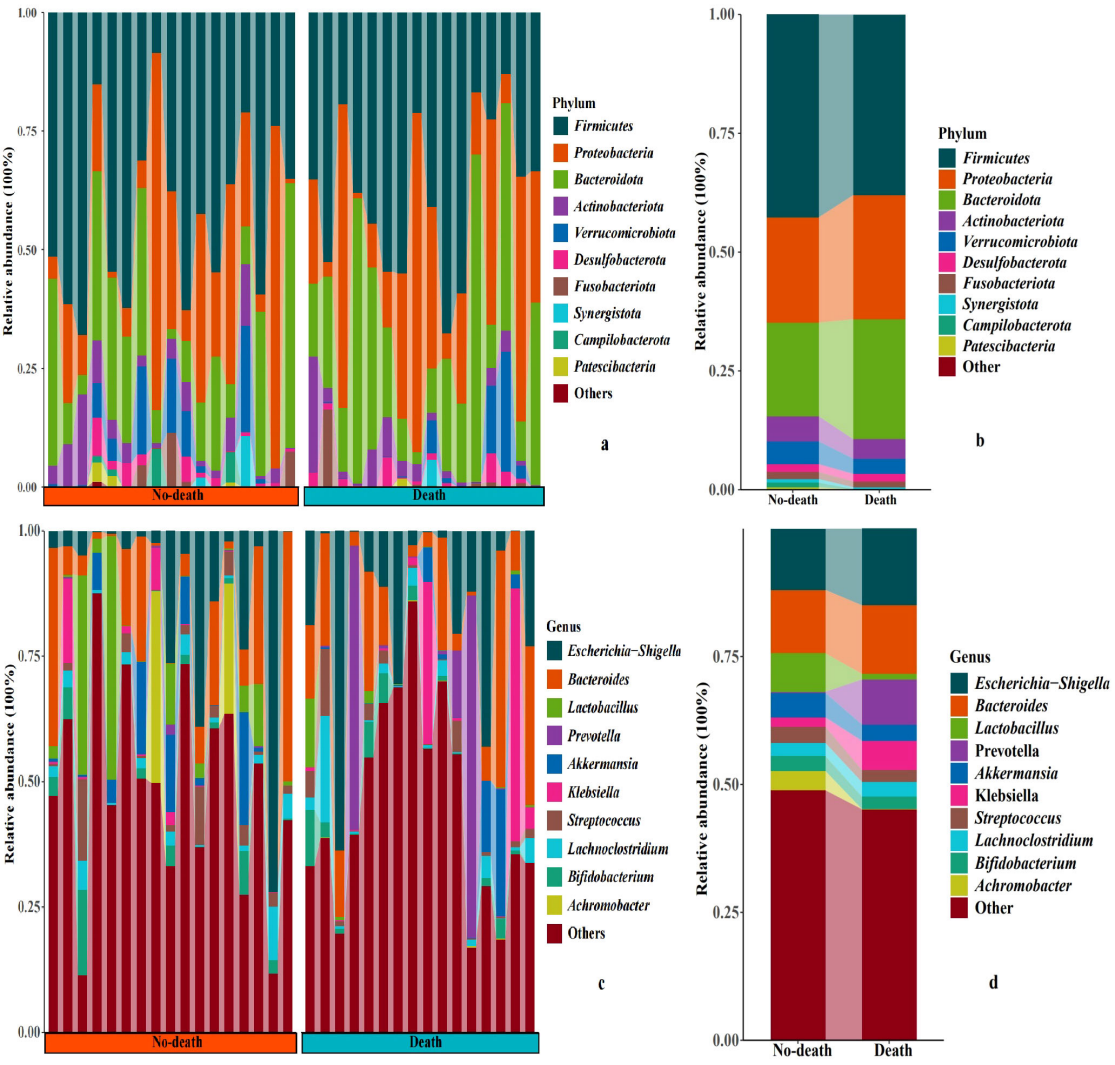
Relative abundance of microbial communities at phyla and genus levels. **(a-d)** histogram shows the average relative abundance of intestinal flora in the death and no-death groups.

The analysis of intestinal microbiome in the death and no-death groups revealed that at the phylum level, Firmicutes, Proteobacteria, Bacteroidota, and Actinobacteriota constitute the predominant components of both groups (>89%) (**Figures 1a, b**). At the genus level, the death and no-death groups are primarily comprised of *Escherichia-Shigella*, *Bacteroides*, *Lactobacillus*, and *Prevotella* (>32%) (**Figures 1c, d**). Individuals in the no-death group exhibited a higher abundance of *Lactobacillus* compared to those in the death group (0.075 vs. 0.011, *p*=0.019). Conversely, patients in the death group demonstrated a higher relative abundance of *Prevotella* (0.088 vs. 0.002, *p*<0.001) and a greater prevalence of *Escherichia* (0.149 vs. 0.120, *p*=0.057) in comparison to individuals in the no-death group.

**Figure 2:**
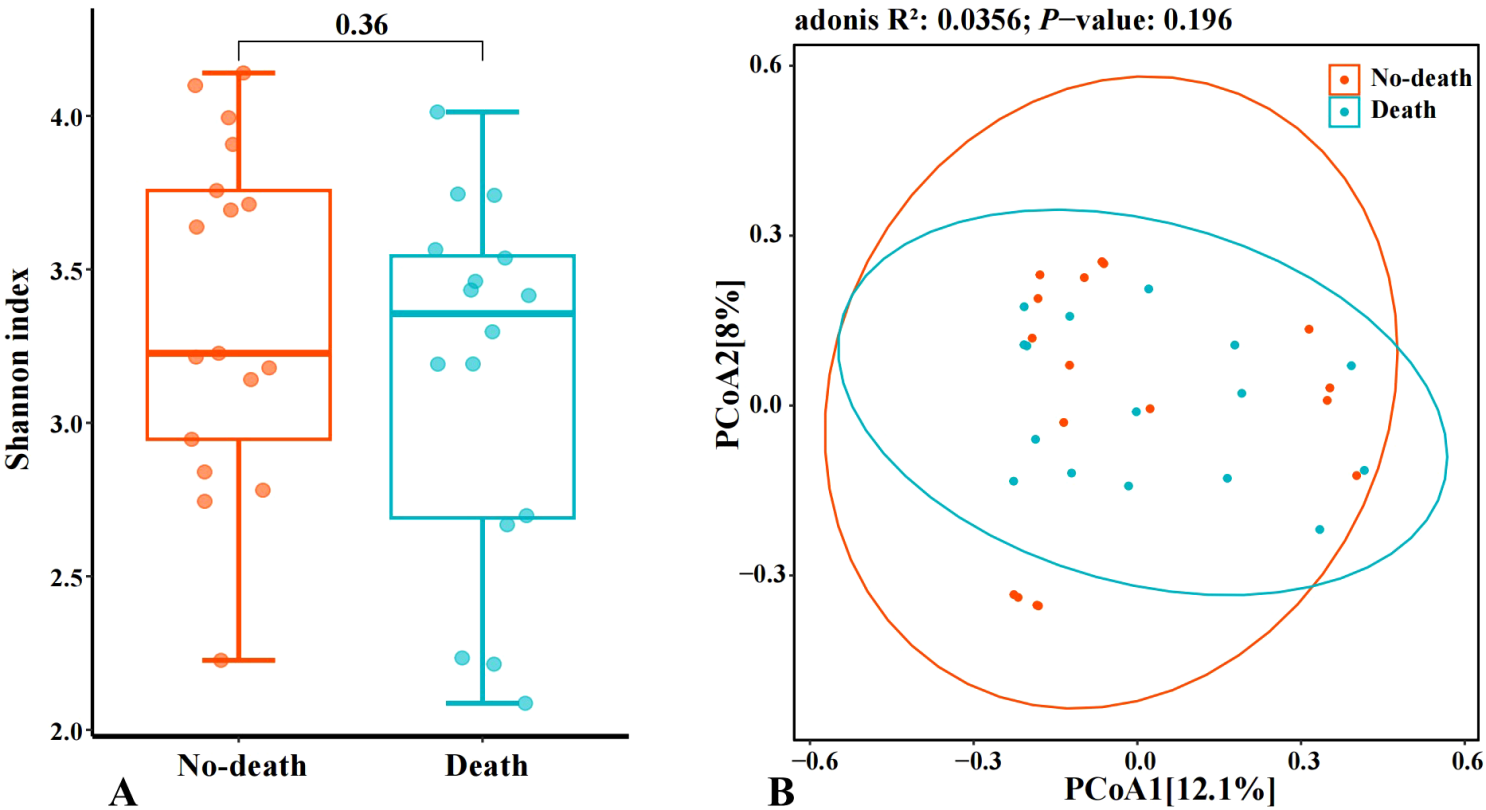
Analysis of changes in intestinal microbiome diversity. (**A**: Comparison of Alpha diversity index of intestinal microbiome among samples; **B**: Comparison of *β*-diversity index of intestinal microbiome among samples).

There were no statistically significant differences in diversity indices between the two groups in terms of alpha and beta diversity. However, it is noteworthy that the median Shannon index of the gut microbiome was higher in the Death group compared to the No-death group (**Figure 2a**). While principal coordinate analysis (PCoA) utilizing the Bray-Curtis distance algorithm did not reveal significant disparities in the spatial distribution distances of the gut microbiome between the two groups, a notable observation was made regarding the Death group samples appearing more clustered. This observation may provide valuable insights for the subsequent analysis of the gut microbiome characteristics in the Death group. (**Figure 2b**). The aforementioned findings suggest that assessments focused on diversity have a constrained effect on differentiating between the two groups. Consequently, we proceeded to conduct a more in-depth analysis of the microbial composition.

In addition, to evaluate whether clinical parameters might confound microbial community structure, we conducted PERMANOVA (Adonis) analyses for all variables listed in **Table 1**. None of these variables reached statistical significance in explaining variation in *β*-diversity (all *p* > 0.05, **Supplementary Figure S1**), indicating that observed microbiome differences were not driven by any single clinical factor.

### Examination of the composition of intestinal microbiome

**Figure 3:**
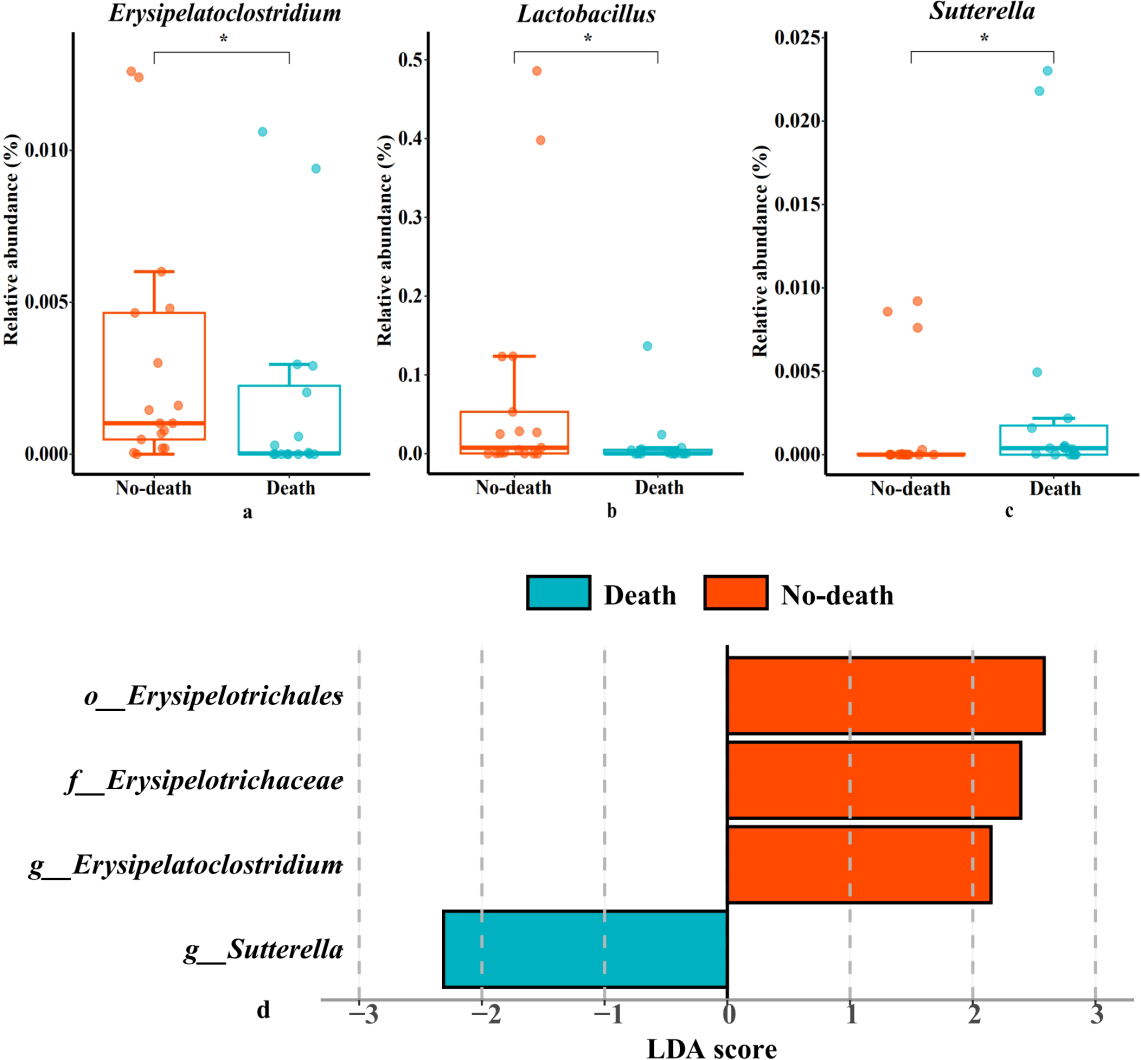
Species with significant differences in two groups and the histogram of LDA value distribution. **(a–c)**: Species with significant differences in two groups. **(d)**: LefSe analysis with linear discriminant analysis (LDA) for bacterial species. Threshold for statistical signifcance: LDA>2log. *p*<0.05 (*) and *p*<0.01 (**).

The outcomes of our research indicated significant differences at the genus level between the death and no-death groups for the species *Erysipelatoclostridium*, *Lactobacillus*, and *Sutterella* (**Figures 3a–c**). Additionally, the results of the LefSe revealed that *Erysipelatoclostridium* exhibited relatively higher abundance in the no-death group, whereas the death group showed higher relative abundance of *Sutterella* (**Figure 3d**). This indicates that *Erysipelatoclostridium* and *Sutterella* may function as distinct microbial biomarkers differentiating the no-death group from the death group, respectively.

### Analysis of risk factors related to mortality

All relevant independent risk factors involved in **Table 1**, the *α*-diversity of the two microbiota groups, as well as the abundances of *Erysipelatoclostium* and *Sutterella* were included in a univariate COX analysis. The number of chronic comorbidities, ACEI/ARB usage, presence of renal/liver/pancreatic diseases, CIRS-G score, and creatinine levels were found may to be associated with mortality at 18 months (**Table 2**). Subsequent COX multivariate analysis revealed that both CIRS-G score and creatinine levels were independently correlated with 18-month mortality (HR: 1.323, 95% CI [1.165-1.502], *p*<0.001 and HR: 1.007, 95% CI [1.002-1.011], *p*=0.004, respectively) (**Table 2**).

**Table 2:** Cox regression analysis of risk factors associated with 18-month mortality in patients (n =33).

Candidate variables	Univariate		Multivariate	
	HR (95%CI)	*p* value	HR (95%CI)	*p* value
Number of chronic comorbidities	1.502 (1.070-2.11)	0.019	–	NS
ACEI/ARB	0.295 (0.106-0.822)	0.020	–	NS
Renal/liver/pancreatic diseases	4.042 (1.432-11.408)	0.008	–	NS
CIRS-G Score	1.277 (1.146-1.423)	<0.001	1.323 (1.165-1.502)	<0.001
Creatinine	1.004 (1.000-1.008)	0.04	1.007 (1.002-1.011)	0.004

NS, not significant.

To assess the robustness of the Cox regression model under small sample conditions, we performed internal validation using bootstrap resampling (500 iterations). Both a reduced model (including CIRS-G Score and creatinine) and a full model (including five significant predictors: Number of chronic comorbidities, CIRS-G Score, ACEI/ARB, creatinine, and Renal/liver/pancreatic diseases) were evaluated. For each model, we calculated the bootstrap-based mean coefficient (Mean), standard deviation (SD_boot), 95% BCa confidence intervals, and coefficient coverage (i.e., whether the primary model estimate falls within the bootstrap CI). Additionally, we calculate the c-index using the LOOCV method: c-index=0.706 (SD: 0.111). The results showed bootstrap validation and LOOCV confirmed the robustness found in the research, CIRS-G Score remains a potential prognostic indicators, at the same time, we also noticed that ACEI/ARB, creatinine, Renal/liver/pancreatic diseases, and the influence of Number of chronic comorbidities direction uncertainty, this needs to be further verification in the larger sample studies. The full details are provided in **Supplementary Tables S2**, **S3** and **Supplementary Figure S2**.

**Figure 4:**
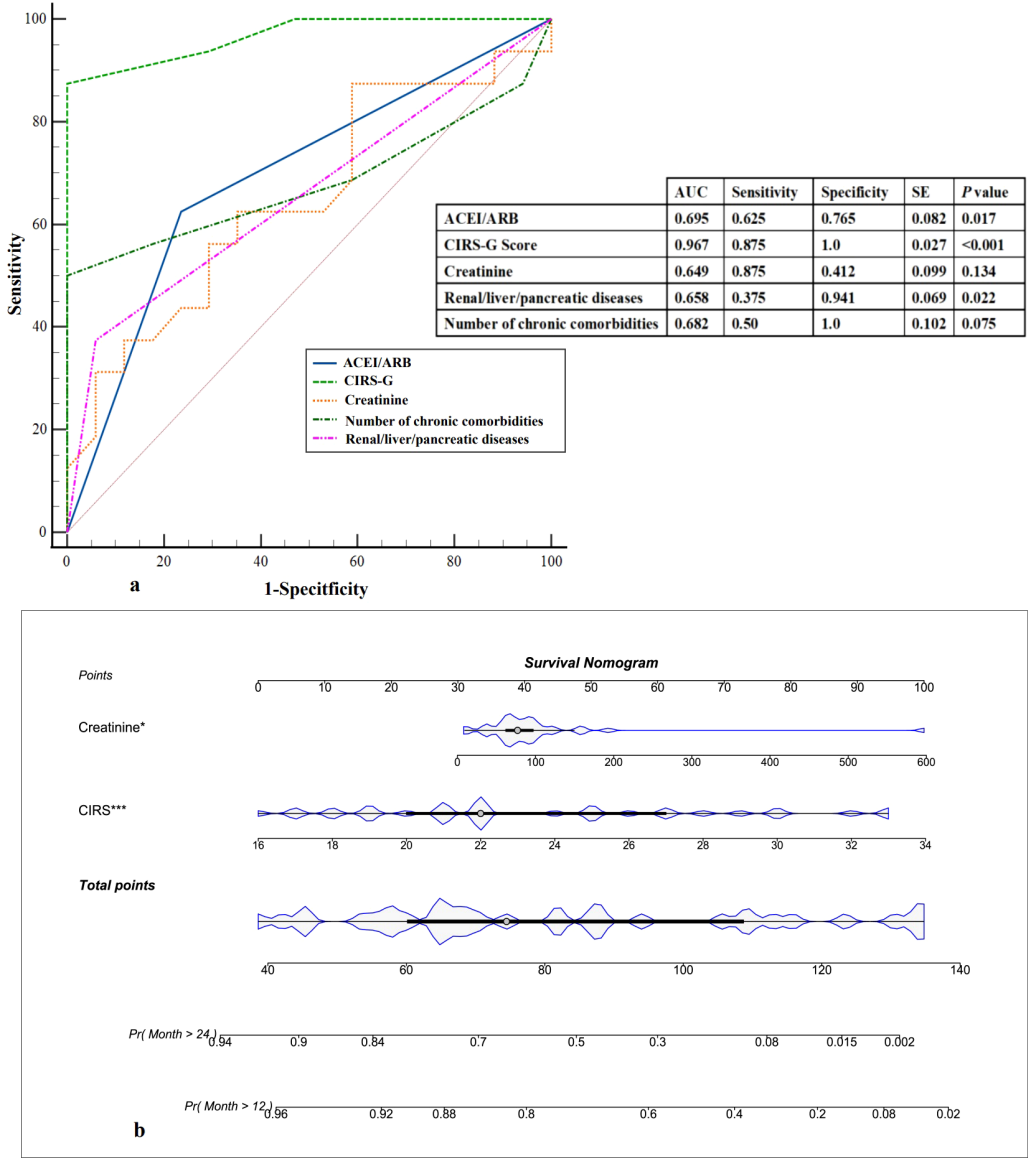
Prediction of 18-month mortality. **(a)** ROC curves. The possibility of the 18-month mortality was measured and compared according to area under the curve values. ROC, receiver operating characteristic curve; AUC, area under the curve. **(b)** Predictive nomogram of 18-month mortality, in which the total score corresponds to a death probability at the bottom.

In the subsequent analysis, we incorporated statistically significant risk factors identified through univariate analysis. The area under the curve (AUC) of the receiver Operating characteristics (ROC) curve was utilized to assess the predictive capability of these factors. The CIRS-G Score emerged as the most crucial clinical indicator for distinguishing between the two groups, exhibiting the highest AUC of 0.967 (**Figure 4a**). However, due to the limited sample size (n=33), the ROC results, including the CIRS-G AUC value, may be influenced by overfitting. These findings should be interpreted with caution and require validation in larger, independent cohorts. Next, nomogram models were constructed to forecast mortality rates at 12 months and 24 months (**Figure 4b**). These findings showed the potential utility of the CIRS-G Score in predicting the 18-month mortality risk in patients with multiple comorbidities and polypharmacy.

### Correlation between the intestinal microbiome and clinical variables

**Figure 5:**
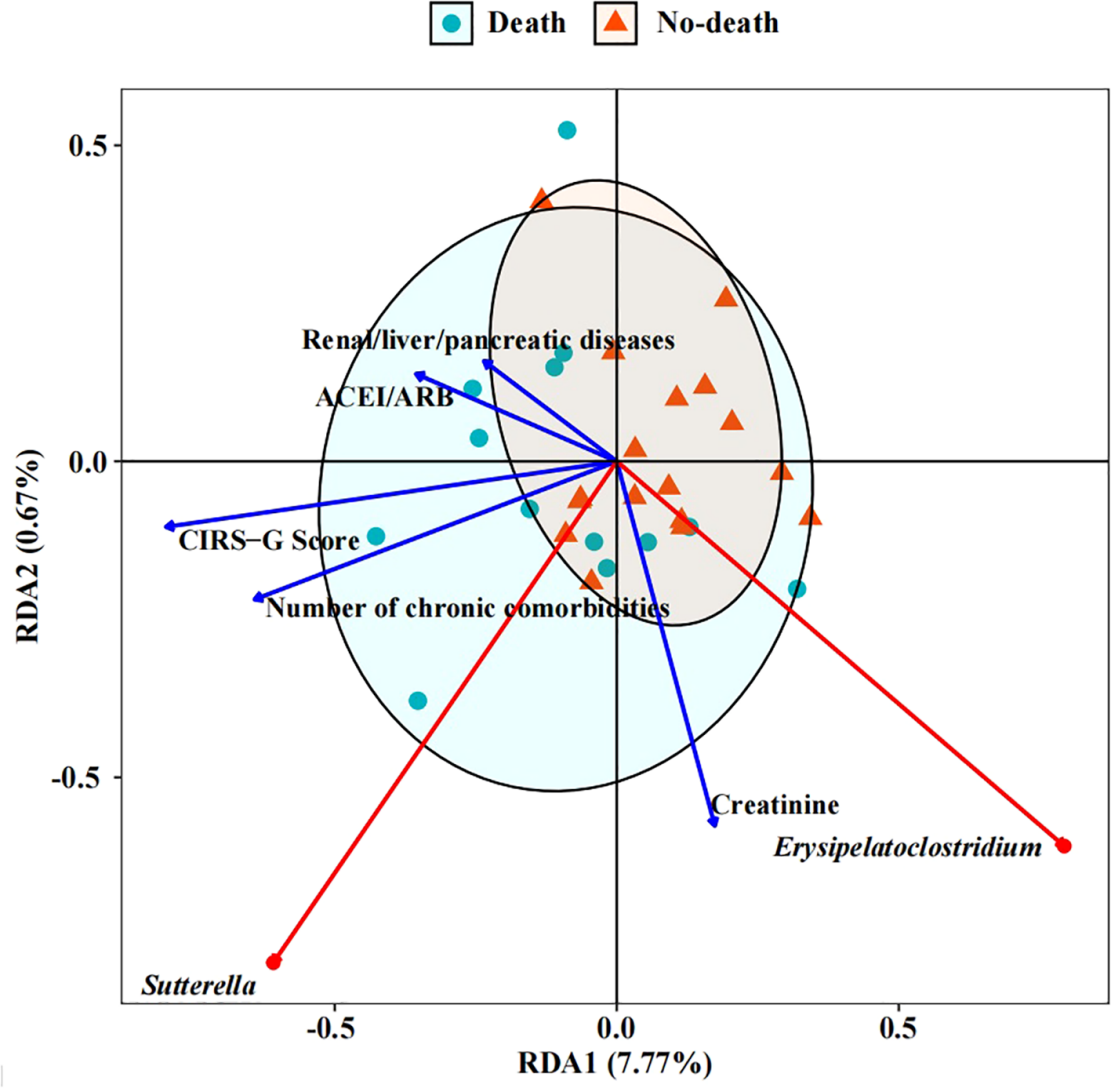
RDA (Redundancy analysis) of intestinal microbiome with clinical indicators. The length of the factor arrow can represent the influence of the factor on the intestinal microbiome. The angles between the arrows represent positive and negative correlations.

The outcomes of the RDA analysis reveal a close association between *Erysipelatoclostium* and *Sutterella* with specific clinical parameters such as the number of chronic comorbidities, ACEI/ARB usage, and the presence of renal, liver, or pancreatic diseases, as well as the CIRS-G Score and Creatinine levels (**Figure 5**). *Erysipelatoclostridium*, which is more abundant in the no-death group, exhibited a negative correlation with CIRS-G score, ACEI/ARB usage, number of chronic comorbidities, and renal/liver/pancreatic diseases. Conversely, *Sutterella*, significantly enriched in the death group, showed a positive correlation with the aforementioned clinical parameters. Furthermore, a positive correlation was observed between creatinine levels and both genera. These findings suggest a potential correlation between intestinal microbiome composition and the clinical characteristics of patients, providing insights into the overall health status of the patients to a certain extent.

## Discussion

This study examines the impact of intestinal microbiome and disease on mortality in a cohort of long-living male patients with multiple comorbidities and polypharmacy. The findings suggest that mortality risk is influenced by the presence of multiple diseases and a high CIRS-G Score. Furthermore, specific species of intestinal microbiome show correlations with key clinical parameters. For instance, a negative correlation was observed between CIRS-G Score and *Erysipelatoclostridium*, while a positive correlation was found with *Sutterella*. These results suggest that there may be a potential link between intestinal microbiome composition and clinical outcomes, which could be significant for the 18-month survival of patients.

Factors such as age, gender, and environment can influence the composition of intestinal microbiome. Research indicates that drug use ranks as the third most influential factor after age and gender. Among various drugs, PPI, laxatives, metformin, and antibiotics are reported to have the most significant impact on the intestinal microbiome (Vich Vila et al., 2020; Weersma et al., 2020; Nagata et al., 2022). Current research predominantly focuses on the intestinal microbiome alterations associated with antibiotic treatment or single non-antibiotic drug use, with limited studies exploring the effects of non-antibiotics and multi-drug regimens on intestinal microbiome. To minimize potential confounding variables, our study included drugs commonly used by patients known to have substantial effects on intestinal microecology. Our findings revealed no significant correlation between the number of drugs used and patient mortality at 18 months (*p*=0.939). A comprehensive analysis utilizing COX multivariables, ROC curves, and nomograms identified the CIRS-G Score as the most robust independent risk factor for increased mortality risk in patients. This observation aligns with previous research indicating that the CIRS-G Score is valuable in identifying heightened mortality risk in senior cancer patients (Canoui-Poitrine et al., 2022; Benderra et al., 2023). Additionally, Benraad et al. demonstrated that the CIRS-G Score can independently predict outcomes (death or transfer to a higher-level hospital) in senior patients hospitalized for acute psychiatric conditions (Benraad et al., 2018). These studies underscore the significant predictive value of the CIRS-G Score in clinical prognosis and outcomes. Nonetheless, the study’s limited sample size necessitates further expansion for more comprehensive evaluation.

The complexity and severity of diseases tend to increase with age in the senior population, often necessitating multi-drug therapy. It has been reported that the impact of multi-drug therapy on the intestinal microecology of patients may be more significant that of coexisting multiple diseases (Nagata et al., 2022). However, our study suggests that the status of the disease plays a more crucial role in patient clinical outcomes than the medications administered. Severe disease status may be linked to decreased reduced microbiota diversity, reduced probiotics, and increased pathogen abundance (Victoria et al., 2022). Our findings indicate that the *α*-diversity in the intestinal microbiome of patients in the no-death group was slightly higher than in the death group, consistent with the results reported by Andrea et al (Ticinesi et al., 2017). Our results revealed that the predominant phylum in the intestinal microbiome of long lived patients were Firmicutes, Proteobacteria, and Bacteroidetes, while at the genus level, *Escherichia-Shigella* and *Bacteroides* were most prevalent. These findings align with research conducted on critically ill patients (Oami et al., 2019; Zhou et al., 2023). Alterations in the composition of the intestinal microbiome may result in dysbiosis, a condition that has been linked to a variety of diseases, including inflammatory bowel disease, obesity, diabetes, and numerous age-related ailments (Zhang et al., 2015; Sokol et al., 2017; Ling et al., 2022; Chen et al., 2023). Our study revealed that the relative abundance of *Lactobacillus* and *Erysipelatoclostridium* was significantly higher in the no-death group compared to the death group, whereas the relative abundance of *Sutterella* was higher in the death group. *Lactobacillus*, a prevalent microorganism in the human gut known for its health benefits (Klaenhammer and Azcarate-Peril, 2007; Wang et al., 2021), *Lactobacillus* can safeguard the body from sepsis-induced organ damage by restoring the gut microbiome (Han et al., 2022). Conversely, *Erysipelatoclostridium*, which demonstrated a negative correlation with CIRS-G scores, has been shown to facilitate the production of beneficial short-chain fatty acids, such as butyrate, which may contribute to enhanced intestinal health (Estaki et al., 2016; Liu et al., 2019; Xiao et al., 2021). In contrast, *Sutterella*, which was enriched in the death group, exhibited a mild pro-inflammatory capacity and may potentially play a role in the development of antibiotic-associated diarrhea and other gastrointestinal diseases (Moon et al., 2015; Hiippala et al., 2016). The study population had a high burden of chronic diseases and polypharmacy, and the impact of these conditions on the composition of the gut microbiota may not be fully measured and explained by the indicators considered. For the same reason, the sample size may not be sufficient to adequately represent the high inter-individual variability of the microbiota, which may affect the results.

*Erysipelatoclostridium* is a gram-positive bacterium that belongs to *Erysipelotrichaceae*, a group widely distributed in nature and known to parasitize mammals, birds, and fish. A recent comprehensive predictive and validation study conducted by Nguyen et al. (2023) demonstrated that a high abundance of *Erysipelatoclostridium* was linked to reduced all-cause mortality in patients undergoing allogeneic hematopoietic cell transplantation. Other research has indicated that the butyrate-producing strain of *Erysipelotrichaceae* has significantly decreased in the feces of children with autism spectrum disorder (Liu et al., 2019; Xiao et al., 2021). Butyric acid is a short-chain fatty acid associated with improved intestinal health. A study on cardiorespiratory fitness revealed that increased levels of butyric acid were correlated with a rise in the abundance of *Erysipelotrichaceae* species, suggesting a potential role for these bacteria in promoting overall health and disease resistance (Estaki et al., 2016). Furthermore, *Erysipelotrichaceae* has been reported to be beneficial in the treatment response or health maintenance of patients with hepatobiliary and colon cancer (Loman et al., 2019; Mao et al., 2021). However, Xu et al. (2019) demonstrated that *Erysipelotrichaceae* was associated with increased mortality in patients in neurological intensive care units. Yu et al. (2023) identified *Erysipelatoclostridium* as a potential pathogen associated with colonic disease, with its abundance significantly increased in mice with IBD. These conflicting finding suggest that *Erysipelatoclostridium* may have varying roles in different clinical settings. LefSe analysis in this study revealed that *Erysipelatoclostridium* was the predominant bacterium in no-death patients. Additionally, *Erysipelatoclostridium* showed a negative correlation with CIRS-G Score, ACEI/ARB, number of chronic comorbidities, and renal/liver/pancreatic diseases. Notably, the correlations with CIRS-G Score and number of chronic comorbidities were more pronounced compared to other parameters. This implies that in the studied patient population, elevated relative abundance of *Erysipelatoclostridium* may be associated with improved overall health and a reduced risk of mortality. This association may be attributed to the advantageous effects of its metabolites, such as butyrate, on gut barrier function and immune response. Nevertheless, it is crucial to emphasize that in this study, the patients in the non-death group received multiple drug treatments, and their final quality of life was also found to be unsatisfactory. Consequently, further research is necessary to comprehensively elucidate the mechanisms underpinning these relationships.

Members of the genus *Sutterella* are significant symbiotic bacteria in the gut, with a high population in the duodenum of healthy adults that gradually decreases towards the colon. These strains have shown mild pro-inflammatory capabilities in the human gastrointestinal tract (Hiippala et al., 2016). In a mouse model, it has been observed that a mother mouse can transmit susceptibility to gut diseases to her offspring through *Sutterella* (Moon et al., 2015). Studies have indicated that *Sutterella* may have a crucial role in the development of Antibiotic-associated diarrhea (AAD) (Lv et al., 2017). Furthermore, the abundance of *Sutterella* is elevated in gastrointestinal disorders in children with autism spectrum disorder (Williams et al., 2012; Wang L et al., 2013). In our investigation, *Sutterella* was found to be significantly enriched in the death group and positively correlated with CIRS-G scores. This suggests that a higher relative abundance of this genus may be associated with worse clinical outcomes, possibly due to its proinflammatory effects. This association highlights the importance of considering *Sutterella* as a potential biomarker for predicting patient outcomes and emphasizes the need for further studies to explore its exact role in disease progression and its potential as a therapeutic target.

Despite efforts to mitigate the impact of confounding variables, this study has inherent limitations. The study population exhibits a significant prevalence of chronic diseases and disabilities, the influence of which on intestinal microbiome composition may not be comprehensively acknowledged and elucidated by the variables under consideration. Moreover, the small sample size may not sufficiently capture the substantial variability in intestinal microbiome composition among individuals. Consequently, this investigation serves as a preliminary exploratory study. Future research endeavors should not only enlarge the sample size but also ascertain whether alterations in fecal microbiota composition among long-living patients with high-burden geriatric syndromes are influenced by medication, the sudden onset of chronic illnesses, or the use of antibiotics.

Additionally, the absence of positive and negative controls, as well as biological replicates, constitutes an inherent limitation of this study. This may limit the ability to discriminate true biological signals from potential contaminants such as kitome and splashome artifacts. Future studies should incorporate appropriate control samples and technical replicates to increase the reliability and interpretability of microbial sequencing data.

## Conclusion

The assessment of survival status in elderly patients with multiple comorbidities and polypharmacy suggests that elevated CIRS-G scores and creatinine levels may be associated with increased mortality at 18 months. The CIRS-G score demonstrated potential as a predictive marker for mortality within this cohort and showed a possible association with specific components of the gut microbiota, such as *Erysipelatoclostridium* and *Sutterella*. These findings indicate that the CIRS-G score could serve as a practical tool to identify high-risk older adults who may benefit from closer clinical monitoring and individualized interventions, including considerations of gut microbiota profiles during pharmacological treatment.

However, several limitations must be acknowledged. First, the small sample size limits the statistical power and generalizability of the findings. Second, the presence of multiple and diverse comorbid conditions introduces potential confounding factors, which may independently influence both microbiome composition and clinical outcomes, thereby complicating causal interpretations. Future studies should include larger, more diverse populations and adopt rigorous control designs to validate these preliminary observations and better disentangle the effects of comorbidities, medication use, and microbiome alterations on patient prognosis.

## Data Availability

The requested data are deposited in the Sequence Read Archive under the BioProject, accession number PRJNA1264796.
