# Supplementary material for: Investigating potential links between gut microbiome, clinical parameters, and mortality in long-living male patients receiving multi-drug therapy
Source: Front Cell Infect Microbiol. 2025 Jul 7;15:1456794. doi: 10.3389/fcimb.2025.1456794 (PMC12277382; doi:10.3389/fcimb.2025.1456794)

Table S1. Sequencing data quality control information table.

| SampleID | ReadNum | BaseNum | GC(%) | N(%) | Q20(%) | Q30(%) | Q30(%)>80% | Average Q30(%) | Average Q30(%)>80% | ReadNum>=45000 | Qualified |
| --- | --- | --- | --- | --- | --- | --- | --- | --- | --- | --- | --- |
| A10_R1 | 80,376 | 18,229,315 | 53.34 | 0 | 97.28 | 92.51 | Yes | 92.065 | Yes | Yes | Yes |
| A10_R2 | 80,376 | 18,066,003 | 55.36 | 0 | 96.97 | 91.62 | Yes |  |  |  |  |
| A11_R1 | 81,920 | 18,597,157 | 53.7 | 0 | 97.31 | 92.39 | Yes | 91.54 | Yes | Yes | Yes |
| A11_R2 | 81,920 | 18,427,566 | 55.34 | 0 | 96.62 | 90.69 | Yes |  |  |  |  |
| A12_R1 | 75,766 | 17,154,905 | 52.18 | 0 | 96.79 | 91.39 | Yes | 91.295 | Yes | Yes | Yes |
| A12_R2 | 75,766 | 16,999,293 | 53.79 | 0 | 96.77 | 91.2 | Yes |  |  |  |  |
| A13_R1 | 61,239 | 13,891,971 | 50.42 | 0 | 94.98 | 91.66 | Yes | 87.545 | Yes | Yes | Yes |
| A13_R2 | 61,239 | 13,776,397 | 51.86 | 0 | 88.52 | 83.43 | Yes |  |  |  |  |
| A14_R1 | 92,892 | 21,073,891 | 53.52 | 0 | 96.32 | 93.88 | Yes | 90.185 | Yes | Yes | Yes |
| A14_R2 | 92,892 | 20,883,699 | 54.97 | 0 | 91.11 | 86.49 | Yes |  |  |  |  |
| A16_R1 | 57,589 | 13,071,178 | 52.41 | 0 | 97.77 | 93.98 | Yes | 90.88 | Yes | Yes | Yes |
| A16_R2 | 57,589 | 12,952,952 | 54.89 | 0 | 94.88 | 87.78 | Yes |  |  |  |  |
| A18_R1 | 84,149 | 19,095,836 | 51.21 | 0 | 95.06 | 91.8 | Yes | 91.37 | Yes | Yes | Yes |
| A18_R2 | 84,149 | 18,928,642 | 52.39 | 0 | 94 | 90.94 | Yes |  |  |  |  |
| A19_R1 | 73,944 | 16,777,349 | 51.24 | 0 | 97.65 | 93.54 | Yes | 92.295 | Yes | Yes | Yes |
| A19_R2 | 73,944 | 16,634,065 | 51.45 | 0 | 96.81 | 91.05 | Yes |  |  |  |  |
| A1_R1 | 55,809 | 12,666,378 | 49.2 | 0 | 97.76 | 93.68 | Yes | 92.58 | Yes | Yes | Yes |
| A1_R2 | 55,809 | 12,555,119 | 51.61 | 0 | 96.97 | 91.48 | Yes |  |  |  |  |
| A20_R1 | 87,488 | 19,842,620 | 53.43 | 0 | 96.33 | 93.9 | Yes | 90.575 | Yes | Yes | Yes |
| A20_R2 | 87,488 | 19,661,912 | 54.61 | 0 | 91.49 | 87.25 | Yes |  |  |  |  |
| A21_R1 | 81,358 | 18,457,391 | 52.42 | 0 | 97.82 | 93.77 | Yes | 91.92 | Yes | Yes | Yes |
| A21_R2 | 81,358 | 18,294,463 | 53.41 | 0 | 96.33 | 90.07 | Yes |  |  |  |  |
| A22_R1 | 88,504 | 20,089,388 | 52.31 | 0 | 96.58 | 94.24 | Yes | 94.39 | Yes | Yes | Yes |
| A22_R2 | 88,504 | 19,910,613 | 54.06 | 0 | 96.42 | 94.54 | Yes |  |  |  |  |
| A25_R1 | 87,610 | 19,882,846 | 50.89 | 0 | 96.88 | 91.06 | Yes | 90.015 | Yes | Yes | Yes |
| A25_R2 | 87,610 | 19,705,915 | 52.37 | 0 | 96 | 88.97 | Yes |  |  |  |  |
| A27_R1 | 74,999 | 17,016,437 | 53.29 | 0 | 94.6 | 90.78 | Yes | 90.425 | Yes | Yes | Yes |
| A27_R2 | 74,999 | 16,868,053 | 53.27 | 0 | 93.57 | 90.07 | Yes |  |  |  |  |
| A28_R1 | 78,931 | 17,914,284 | 53.79 | 0 | 94.78 | 90.95 | Yes | 90.275 | Yes | Yes | Yes |
| A28_R2 | 78,931 | 17,755,956 | 56.8 | 0 | 93.45 | 89.6 | Yes |  |  |  |  |
| A29_R1 | 74,689 | 16,951,160 | 53.95 | 0 | 97.14 | 92.12 | Yes | 91.85 | Yes | Yes | Yes |
| A29_R2 | 74,689 | 16,800,967 | 54.97 | 0 | 97 | 91.58 | Yes |  |  |  |  |
| A2_R1 | 66,006 | 14,978,829 | 52.74 | 0 | 97.61 | 93.21 | Yes | 91.65 | Yes | Yes | Yes |
| A2_R2 | 66,006 | 14,848,265 | 53.72 | 0 | 96.34 | 90.09 | Yes |  |  |  |  |
| A30_R1 | 77,749 | 17,644,589 | 53.52 | 0 | 94.53 | 90.95 | Yes | 89.675 | Yes | Yes | Yes |
| A30_R2 | 77,749 | 17,489,072 | 54.27 | 0 | 92.45 | 88.4 | Yes |  |  |  |  |
| A31_R1 | 79,898 | 18,131,584 | 52.03 | 0 | 97.8 | 93.72 | Yes | 92.01 | Yes | Yes | Yes |
| A31_R2 | 79,898 | 17,972,953 | 54.69 | 0 | 96.45 | 90.3 | Yes |  |  |  |  |
| A33_R1 | 105,001 | 23,833,289 | 52.85 | 0 | 97.54 | 93.2 | Yes | 92.02 | Yes | Yes | Yes |
| A33_R2 | 105,001 | 23,621,075 | 54.49 | 0 | 96.6 | 90.84 | Yes |  |  |  |  |
| A34_R1 | 73,240 | 16,623,517 | 50.75 | 0 | 95.32 | 92.3 | Yes | 91.45 | Yes | Yes | Yes |
| A34_R2 | 73,240 | 16,477,227 | 52.91 | 0 | 93.78 | 90.6 | Yes |  |  |  |  |
| A35_R1 | 85,153 | 19,328,134 | 52.61 | 0 | 97.57 | 93.43 | Yes | 92.295 | Yes | Yes | Yes |
| A35_R2 | 85,153 | 19,154,872 | 53.88 | 0 | 96.81 | 91.16 | Yes |  |  |  |  |
| A36_R1 | 88,216 | 20,026,035 | 52.9 | 0 | 97.53 | 93.45 | Yes | 91.8 | Yes | Yes | Yes |
| A36_R2 | 88,216 | 19,844,673 | 55.38 | 0 | 96.32 | 90.15 | Yes |  |  |  |  |
| A37_R1 | 93,168 | 21,149,357 | 51.64 | 0 | 97.53 | 93.38 | Yes | 92.31 | Yes | Yes | Yes |
| A37_R2 | 93,168 | 20,960,867 | 52.54 | 0 | 96.8 | 91.24 | Yes |  |  |  |  |
| A39_R1 | 95,454 | 21,656,284 | 52.94 | 0 | 94.97 | 91.25 | Yes | 90.59 | Yes | Yes | Yes |
| A39_R2 | 95,454 | 21,466,675 | 53.01 | 0 | 93.44 | 89.93 | Yes |  |  |  |  |
| A3_R1 | 81,060 | 18,396,975 | 51.15 | 0 | 94.57 | 90.98 | Yes | 90.67 | Yes | Yes | Yes |
| A3_R2 | 81,060 | 18,235,418 | 52.24 | 0 | 93.51 | 90.36 | Yes |  |  |  |  |
| A40_R1 | 75,923 | 17,232,217 | 49.69 | 0 | 94.25 | 90.61 | Yes | 89.83 | Yes | Yes | Yes |
| A40_R2 | 75,923 | 17,080,667 | 53.19 | 0 | 92.79 | 89.05 | Yes |  |  |  |  |
| A41_R1 | 70,233 | 15,927,711 | 50.11 | 0 | 97.72 | 94.79 | Yes | 93.13 | Yes | Yes | Yes |
| A41_R2 | 70,233 | 15,788,046 | 51.35 | 0 | 95.68 | 91.47 | Yes |  |  |  |  |
| A4_R1 | 63,008 | 14,301,371 | 53.59 | 0 | 97.44 | 92.78 | Yes | 90.14 | Yes | Yes | Yes |
| A4_R2 | 63,008 | 14,172,396 | 56.2 | 0 | 95.2 | 87.5 | Yes |  |  |  |  |
| A6_R1 | 83,703 | 18,996,769 | 53.55 | 0 | 97.88 | 93.83 | Yes | 91.735 | Yes | Yes | Yes |
| A6_R2 | 83,703 | 18,827,940 | 56.16 | 0 | 96.22 | 89.64 | Yes |  |  |  |  |
| A7_R1 | 69,750 | 15,831,924 | 53.63 | 0 | 97.71 | 93.21 | Yes | 92.405 | Yes | Yes | Yes |
| A7_R2 | 69,750 | 15,689,957 | 55.36 | 0 | 97.01 | 91.6 | Yes |  |  |  |  |
| A8_R1 | 71,214 | 16,166,794 | 50.57 | 0 | 97.69 | 93.5 | Yes | 93.155 | Yes | Yes | Yes |
| A8_R2 | 71,214 | 16,021,365 | 51.42 | 0 | 97.52 | 92.81 | Yes |  |  |  |  |
| A9_R1 | 83,935 | 19,054,246 | 49.32 | 0 | 97.65 | 93.44 | Yes | 93.295 | Yes | Yes | Yes |
| A9_R2 | 83,935 | 18,883,919 | 49.31 | 0 | 97.63 | 93.15 | Yes |  |  |  |  |

Table S2. Bootstrap estimates (n = 500) for the full Cox regression model.

| Variable | Mean | SD_boot | CI_lower | CI_upper | Coverage |
| --- | --- | --- | --- | --- | --- |
| Number of chronic comorbidities | -0.1174 | 0.267 | -0.7926 | 0.4579 | 1 |
| CIRS-G Score | 0.2863 | 0.0843 | 0.0828 | 0.3484 | 1 |
| ACEI/ARB | -0.6215 | 1.1164 | -1.6945 | 0.8389 | 1 |
| Creatinine | -0.0018 | 0.008 | -0.015 | 0.0228 | 1 |
| Renal/liver/pancreatic diseases | 1.0858 | 1.2818 | -2.4852 | 2.3308 | 1 |

Table S3. Bootstrap estimates (n = 500) for the reduced Cox regression model.

| Variable | Mean | SD_boot | CI_lower | CI_upper | Coverage |
| --- | --- | --- | --- | --- | --- |
| CIRS-G Score | 1.2133 | 0.1938 | 0.8042 | 1.4564 | 1 |
| Creatinine | 0.3628 | 0.2721 | -0.5467 | 0.7128 | 1 |

Table S4. The result of the Variance Inflation Factor (VIF)

| Variable | vif_values |
| --- | --- |
| Number of chronic comorbidities | 2.088 |
| CIRS-G Score | 1.427 |
| ACEI/ARB | 1.111 |
| Creatinine | 1.907 |
| Renal/liver/pancreatic diseases | 2.621 |

Figure S1. Results of PERMANOVA (Adonis) analyses for all clinical and demographic variables listed in Table 1. R² values represent the proportion of variance in microbiome *β*-diversity explained by each variable. No variables reached statistical significance (all *p* > 0.05).


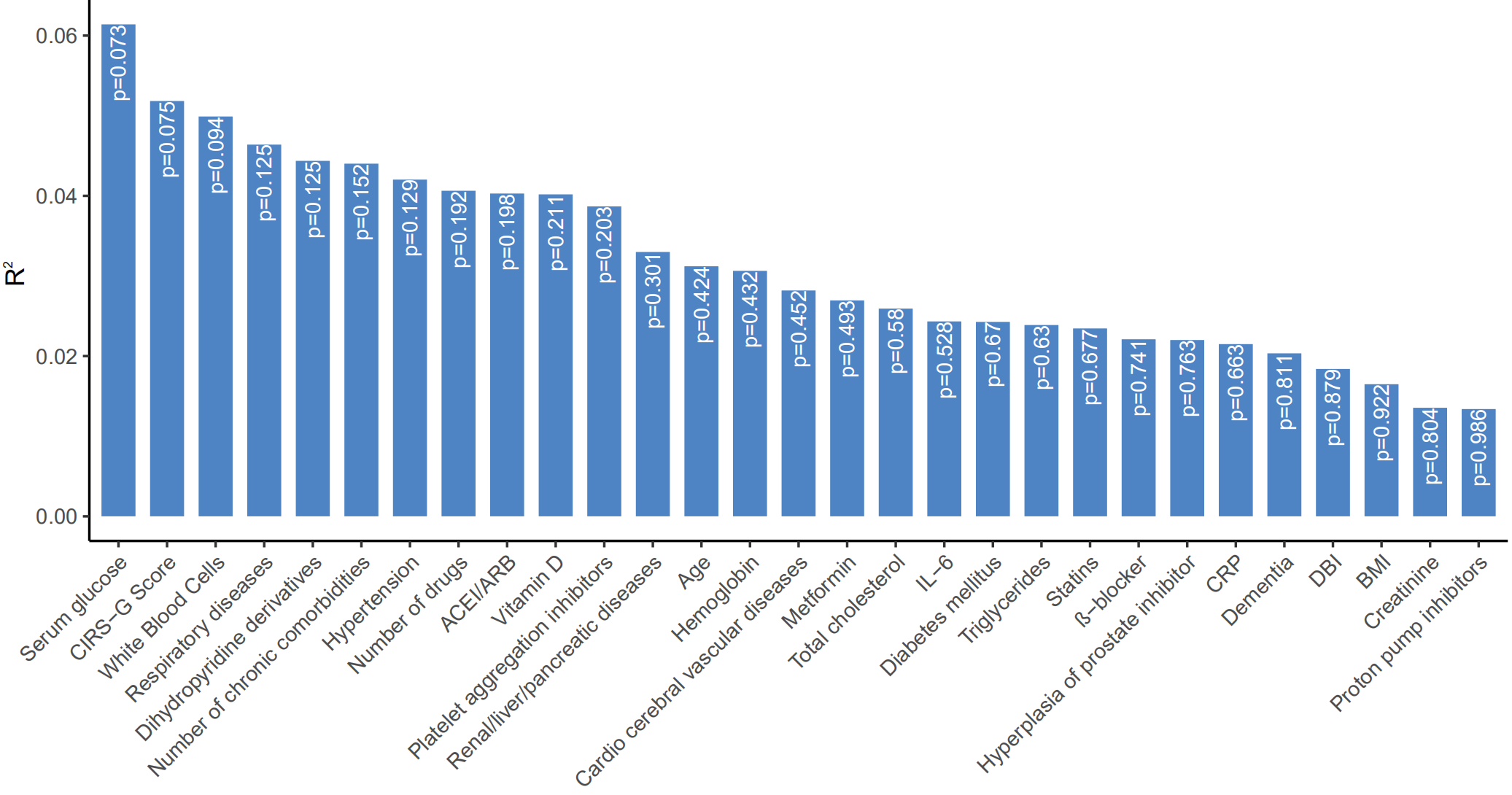


Figure S2. Bootstrap estimates (n = 500) for the full and the reduced 95% confidence intervals.


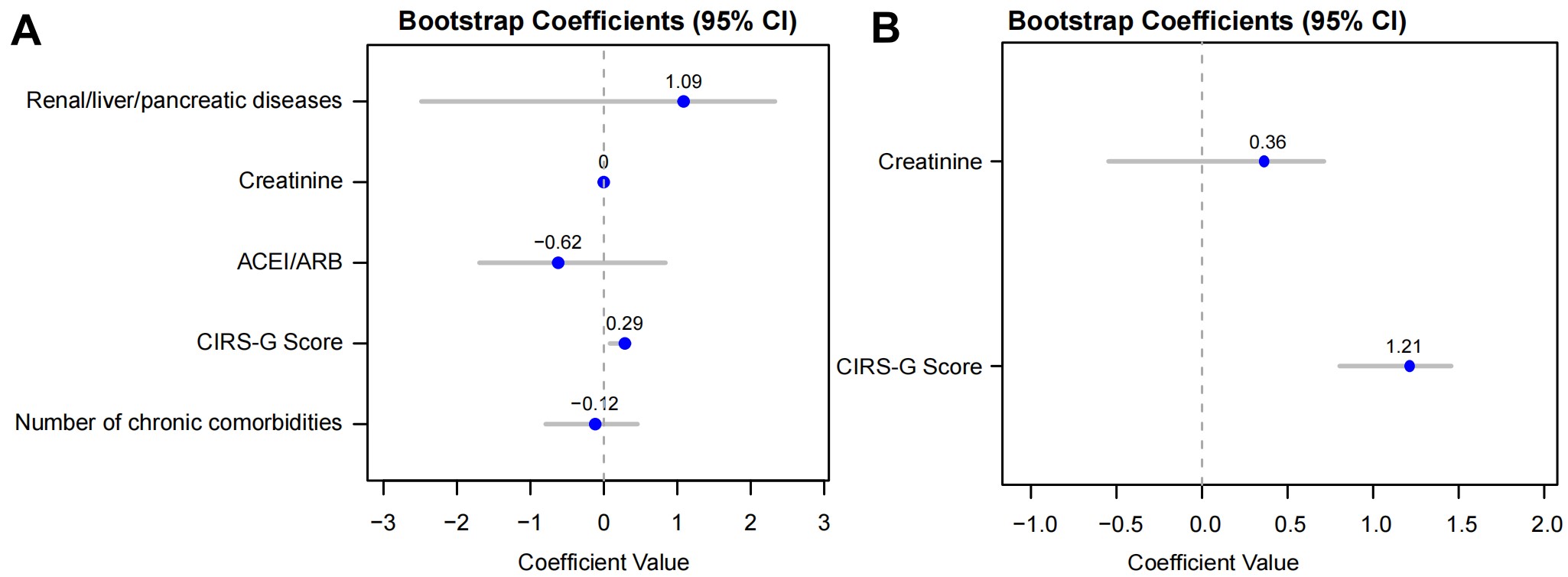

Supplement: Supplementary file 1 [file DataSheet1.docx]
